# THE EFFECT OF MEDIA USAGE DURING THE COVID-19 PANDEMIC ON DEPRESSIVE SYMPTOMS IN KOREAN OLDER ADULTS

**DOI:** 10.1093/geroni/igad104.2153

**Published:** 2023-12-21

**Authors:** Miri Kim, Soondool Chung

**Affiliations:** Ewha Womans University, Seoul, Republic of Korea; Ewha Womans University, Seoul, Republic of Korea

## Abstract

The current study was conducted to examine the structural relationship between change in the media usage during the COVID-19 pandemic, media use frequency, perception of social connectivity and depressive symptoms among older people in South Korea. The study sought to address the increased use of media among older adults during the pandemic. A cross-sectional dataset with 398 older adults aged 65 to 94 living in Seoul was analyzed using a structural equation model with the bootstrapping method. The findings are as follows. In terms of direct effects, older adults who used more media during the COVID-19 pandemic showed lower level of depressive symptoms (β=-.141,p<.001), older adults who used media more frequently showed an increase in the perception of social connectivity (β=.123,p<.001), and older adults with higher perception of social connectivity showed lower level of depressive symptoms (β=-.371,p<.001). In terms of the indirect effects, the perception of social connectivity had a significant mediating effect in the relationship between change in the media usage during the COVID-19 pandemic and depressive symptoms (95% CI: -0.044, -.001), as well as in the relationship between media use frequency and depressive symptoms (95% CI: -.059, -.033). The findings of the study underscore the importance of social connectivity in promoting mental health among older adults during periods of social isolation and highlight the potential benefits of media usage.

